# Obstetric Medicine

**Published:** 1858-05

**Authors:** 


### OBSTETRIC MEDICINE.

On Secondary Affections of the Joints in Puerperal Women. By *William Coulson, Esq.*, Surgeon to, and Lecturer on Surgery at, St. Mary's Hospital; and Consulting Surgeon to the City of London Lying-in Hospital.—Puerperal women are occasionally attacked by a severe form of disease attended by low fever, with effusion of pus into the joints, and almost always terminating in death. Various opinions are entertained respecting the nature and causes of this disease. My object in the following remarks is to show that it is connected with pyæmia.

The local affection occurs under two circumstances, viz. after delivery, at the full period of gestation or after abortion in the earlier months. It is important to include the latter kind of cases, which have not been sufficiently noticed, as they throw great light on the true nature of the disease, and dispose of the theory which would attribute the secondary affections to child-bed fever. The articular disease, as I shall presently show, is merely one of the effects of pyæmia; but it receives certain modifications from the puerperal condition with which it is associated. Even in puerperal women these secondary joint-affections may occur under several states which may be distinguished from each other. They are most commonly developed during the course of puerperal fever, from the third to the sixth day of the complaint. In other cases, they occur after convalescence from an attack of puerperal fever. Lastly, in some other cases they set in after parturition, without the patient having presented any symptoms of puerperal fever.

Although the articular affections are essentially the same under these different circumstances, it is useful to distinguish these circumstances, as they modify the general conditions which precede or accompany the local disease. Thus, when the symptoms of purulent infection of the blood are mixed up with those of true puerperal fever, we have a complex malady which has puzzled some of our best accoucheurs, and which, indeed, it would be impossible to understand, if the same affection did not sometimes occur in puerperal women without puerperal fever.

When the articular affection occurs during the course of puerperal fever, the following train of events is generally observed. For the first three or four days, the ordinary signs of puerperal fever are alone recognized; then some symptoms of phlebitis present themselves; or these symptoms may be so slight as to be overlooked. They are soon followed by a change in the condition of the patient. Severe rigors often usher in this change; the fever increases; the countenance is anxious and sallow; the respiration becomes hurried; there is irregular delirium; and the patient sinks.

In these cases, there are two dangerous maladies, viz. puerperal fever and purulent infection, running their course at the same time; and it is not to be wondered at if the general condition of the patient presents an anomalous appearance, or if it be rendered obscure by the predominance of one set of symptoms over the other.

In another set of cases, this obscurity does not exist. The patient has completely recovered from an attack of puerperal fever, or has not had any attack of that complaint; all the dangers of the puerperal state having apparently passed over. She goes on well for the first week or two; there is no fever; no abdominal pain; no apparent danger of any kind. Suddenly, a severe rigor sets in; this is followed by febrile symptoms, small quick pulse, &c.; or the attack may commence with local symptoms, the general disturbance being scarcely perceptible. These latter cases are very remarkable, and not long ago were mistaken for rheumatism.

The disease, with its local effects and constitutional symptoms, may occur after abortion in the early months. Here there are no symptoms of puerperal fever, properly so called; but there may be some slight symptoms of uterine phlebitis. These are often chronic and obscure cases; yet they proceed and terminate like the former series.

The secondary joint-affections are the same under all these different circumstances. They may be either purulent or non-purulent; articular or periarticular; acute or chronic. These different conditions are found to exist in various cases; but the most common form of attack is acute, of a purulent nature, and occupies the interior of the joint. At other times, though extremely acute, the attack is non-purulent, and confined to the exterior of the joint; while, in several cases, there is pus in the cavity of the joint, without any lesion of the articular tissues.

It is also worthy of remark, that in several cases some of the joints are attacked by purulent inflammation, while other joints in the same subject suffer from simple inflammation with effusion of serum.

The period at which the articular affection sets in is various. In a few cases, it has commenced on the second day after delivery; in many other cases, it does not appear until a few days before death, viz. from the twenty-third to the twenty-fifth day. Generally, however, the joints are attacked between the third and fourteenth days. The knee-joint is most frequently the seat of disease. I have found that it is attacked in one-third of the cases in which the joints have suffered; next comes the wrist-joint; then the ankle, the shoulder, elbow, hip; and, lastly, the smaller joints. In a few cases, the purulent effusion has been confined to the symphysis pubis; but I am inclined to think, from the history of these rare cases, that the suppuration of the pubic joint was primary, not secondary—the inflammatory action having extended from the cellular tissue of the pelvis.

The duration of the joint disease necessarily depends on the duration of the primary affection with which it is connected as an effect. It is not often prolonged beyond a week, but it may last from one to three weeks. In chronic cases the duration may extend to three months. I have published a remarkable case, where death took place on the sixty-fifth day after delivery, and on the fifty-fifth after the attack. Mr. Arnott also mentions a case which was prolonged for forty-four days; but no post-mortem appearances are given.

The local articular symptoms, and the changes discovered after death, present several varieties. In some cases there are no local signs of articular disease; even pain about the joint is absent. After death the cavity contains some pus; the articular tissues are quite healthy. These are exceptional cases. It is very rare not to find some one joint visibly affected, although the rest may have been the seat of passive suppuration.

The local disease commonly appears with the signs of synovial inflammation. The attack is always of an acute nature, commencing with pain more or less severe, which is quickly followed by swelling. The color of the skin remains unchanged, and the swelling is evidently caused by effusion of fluid within or round the joint. When the articular disease sets in, as is often the case, only a few days before death, it may continue with little change to the end; in other cases, the pain and tumefaction disappear altogether before death; or they may leave one joint to attack another.

It is, however, important to remark that, although the pain and swelling may somewhat subside, the disease never passes suddenly from one joint to another, as occurs in true rheumatism. Several large joints may suffer in succession; but, after death, we always find some lesions in or around the joint first attacked. In other cases some change in the color of the skin is found, superadded to the pain and swelling. The skin may be hot and red; it is often of a dusky-red color, especially about the knuckles and wrists. In many of these cases the affection is probably confined to the periarticular tissues; whereas, in ordinary diseases of the joints, the matter which surrounds the joint is derived from its cavity; in secondary diseases the pus hardly ever comes from the interior of the joint. I am acquainted with two cases only in which perforation of the cavity of the joint had taken place. These periarticular effusions, then, may be of two kinds. Sometimes the effusion is serous, and subsides to a considerable degree before death; or pus has been effused into the cellular tissue outside the joint, either with or without symptoms of local inflammation.

I am quite unable to determine the circumstances which give rise to these differences, or to explain why the effusion is purulent in some cases, serous in others; why it takes place now *in* the joint, at another time *outside* it. Moreover, the kind and seat of the effusion bear no relation to the gravity of the case, or to the intensity of the local symptoms.

The joint-affections are frequently accompanied by abscesses in the muscles of the legs and arms, preceded by pain and attended by doughy swellings. When these occur in puerperal women they should always excite attention, for they are too often the forerunner of purulent infection of the blood. The changes discovered after death are purulent effusion into the articular cavity without any alteration of tissue; frequently signs of synovial inflammation with erosion and ulceration of the cartilages; more frequently still purulent or serous infiltrations outside the joints, with abscesses in the neighboring muscles. In no case have the bones been found diseased; in no case, likewise, are the lesions confined to the joints; yet in a few cases the joints and intermuscular tissue have only been affected.

The other lesions are those of purulent infection, viz. secondary deposits in the lungs, liver, &c. The brain has not been found affected, as far as I am aware. Pus is always found either in the veins or lymphatics of the uterus; or there is primary abscess in the walls of the uterus, in the cellular tissue of the pelvis, in the symphysis pubis or elsewhere.

Practitioners are now agreed that the puerperal disease of the joints depends on blood-poisoning. The only question on which difference of opinion exists is as to the nature of the poison. Is it pus? Is it some morbid secretion or putrid element introduced into the blood? My own opinion is that these secondary joint-affections, as well as many others, are caused by purulent poisoning of the blood.

1. In the large majority of cases, purulent phlebitis of the parts originally affected has been observed after death. This holds good especially for cases which occur in connection with puerperal fever. The careful observations of M. Tonellé, at the Lying-in-Hospital in Paris, place the fact beyond doubt, and show that, although these secondary joint-affections and the general symptoms which accompany them never take place without having been preceded by primary suppuration, purulent phlebitis and primary suppuration of the cellular tissue do not necessarily give rise to them. The pus-poisoning and secondary deposits are an occasional, but not a constant effect of the phlebitis and primary abscesses. Thus, in two hundred and twenty-two post-mortem examinations of patients who died from puerperal fever, M. Tonellé found suppuration of the veins or lymphatics of the uterus in one hundred and thirty-four cases; yet of these, ten cases only furnished examples of secondary articular disease.

2. In many cases where pus has not been found in the uterine veins or lymphatics, it has been found in other tissues; and the very few cases related where no pus was found show either that the post-mortem examinations were imperfectly conducted, or that the temporary secretion of pus might have been fairly inferred from the symptoms during life.

3. The presence of vitiated and putrid secretions in the uterus does not account for the disease. It is not produced by retention of the placenta after abortion. It does not occur (unless phlebitis exists) in the form of puerperal fever, which is characterized by putrescence and softening of the uterus. It is not produced by the ingestion of putrid animal substances into the stomach.

4. The disease occasionally occurs in females without any of the accompanying circumstances of the puerperal state. Yet in its course, symptoms, and termination, it does not differ from the form which occasionally accompanies puerperal fever, the only modifications being those which arise from the presence or absence of the puerperal fever itself.

I do not deny the pernicious influence of vitiated secretions; but I maintain that all observation and analogy establish the doctrine that, unless these secretions excite purulent phlebitis, or give rise to primary deposits of pus in some of the tissues, they are not followed by the train of symptoms known under the name of pyæmia.—*British Med. Journ.*, March 6, 1858.

